# A new low-dose multi-phase trauma CT protocol and its impact on diagnostic assessment and radiation dose in multi-trauma patients

**DOI:** 10.1007/s10140-017-1496-4

**Published:** 2017-04-05

**Authors:** Zlatan Alagic, Andreas Eriksson, Erika Drageryd, Sara Rezaei Motamed, Marius C. Wick

**Affiliations:** 1 Functional Unit for Musculoskeletal Radiology, Function Imaging and Physiology, Karolinska University Hospital, Karolinska Vägen Solna, SE-17176 Stockholm, Sweden; 2 General Electrics Healthcare Sverige AB, Danderyd, Sweden; 3 Diagnostic Radiology, Institute for Molecular Medicine and Surgery (MMK), Karolinska Institutet, Stockholm, Sweden

**Keywords:** Trauma CT, CT protocol, Radiation dose, Whole-body CT angiography, ASIR-V, Missed injuries

## Abstract

**Purpose:**

Computed tomography (CT) examinations, often using high-radiation dosages, are increasingly used in the acute management of polytrauma patients. This study compares a low-dose polytrauma multi-phase whole-body CT (WBCT) protocol on a latest generation of 16-cm detector 258-slice multi-detector CT (MDCT) scanner with advanced dose reduction techniques to a single-phase polytrauma WBCT protocol on a 64-slice MDCT scanner.

**Methods:**

Between March and September 2015, 109 polytrauma patients (group A) underwent acute WBCT with a low-dose multi-phase WBCT protocol on a 258-slice MDCT whereas 110 polytrauma patients (group B) underwent single-phase trauma CT on a 64-slice MDCT. The diagnostic accuracy to trauma-related injuries, radiation dose, quantitative and semiquantitative image quality parameters, subjective image quality scorings, and workflow time parameters were compared.

**Results:**

In group A, statistically significantly more arterial injuries (*p* = 0.04) and arterial dissections (*p* = 0.002) were detected. In group A, the mean (±SD) dose length product value was 1681 ± 183 mGy*cm and markedly lower when compared to group B (*p* < 0.001). The SDs of the mean Houndsfield unit values of the brain, liver, and abdominal aorta were lower in group A (*p* < 0.001). Mean signal-to-noise ratios (SNRs) for the brain, liver, and abdominal aorta were significantly higher in group A (*p* < 0.001). Group A had significantly higher image quality scores for all analyzed anatomical locations (*p* < 0.02). However, the mean time from patient registration until completion of examination was significantly longer for group A (*p* < 0.001).

**Conclusions:**

The low-dose multi-phase CT protocol improves diagnostic accuracy and image quality at markedly reduced radiation. However, due to technical complexities and surplus electronic data provided by the newer low-dose technique, examination time increases, which reduces workflow in acute emergency situations.

## Introduction

Traumatic injury is the main cause of death in people younger than 45 years in Europe and a leading cause of mortality, morbidity, and permanent disability [1–4]. Potentially critically injured trauma patients are in need of immediate life-saving interventions, and studies have shown a significant drop in mortality rate if the patient is evaluated within the first hour after injury (“the golden hour”) [5, 6]. Finding accurate trauma-related diagnoses under time pressure in critically injured trauma patients is a great diagnostic challenge. Whole-body computed tomography (WBCT), from the top of the scull to the pubic symphysis, has, since the mid 1990s, been an important diagnostic tool in initial clinical trauma management [6]. The alternative to a WBCT is selective imaging, where certain body parts are scanned based on factors such as findings during the physical examination, visible injuries, and the injury mechanism. It has been shown, however, that WBCT significantly reduces the mortality rate in trauma patients compared to selective imaging and also has an effect on the average length of hospital stay [7, 8]. A single-phase WBCT significantly reduces the overall patient time of stay in the CT suite and reduces unnecessary time loss when transfer to the operating room is needed [8]. The benefits of selective imaging are mainly considered to be a reduction of examination time and radiation dose. Compared to selective imaging, WBCT uses more radiation. Studies have shown that radiation >20 mSv before the age of 40 increases the risk of developing cancer with 1/1000 patients [9–11]. Thus, a considerable high amount of research in emergency radiology has strived to maintain good image quality while lowering radiation doses and several dose reduction CT technologies have recently been developed [12–15]. However, a trauma WBCT protocol that combines multiple intravenous contrast medium (CM) phases together with the latest dose reduction techniques, such as the adaptive statistical iterative reconstruction-V (ASiR-V) algorithm, has not yet been systematically analyzed and used in a clinical research project. As the first level 1 trauma radiology unit in Europe, the Karolinska University Hospital Solna installed the latest generation of 16-cm detector Revolution™ multi-detector CT (MDCT) scanner (GE Healthcare, Milwaukee, WI, USA) in their trauma CT room. The system is equipped with ASiR-V, which can reduce radiation dose up to 82%, while lowering image noise. Until the installation of the Revolution™ CT, our emergency radiology unit utilized, on a 64-slice MDCT (LightSpeed VCT, GE Healthcare, Milwaukee, WI, USA), a polytrauma CT protocol that comprised a native scan of the scull, midface, and cervical spine followed by a single-phase CM-enhanced scan of the thorax and abdomen during a venous CM phase only. The goal of this technique is to quickly acquire the body volume and send the patient back to the emergency room or operation theater in the shortest possible time. However, with the development of new CT technologies, such as the Revolution™ CT, whole-body volumes are acquired in only a few seconds and at significantly lower radiation doses, reducing acquisition times and enabling for even CT examinations at multiple CM phases without time or image quality loss [16]. Moreover, in high-energy polytrauma patients, the prevalence of, e.g., cervical fractures with risk for vascular injuries as well as blunt cervical vascular injuries (BCVIs) causing dissections and arterothrombosis as well as the prevalence of large vessel injuries with active extravasations is relatively high, warranting polytrauma CT protocols that also include image acquisition during an arterial CM phase [17]. Thus, concomitant with the installation of the Revolution™ CT, a modification of the standard single-phase trauma CT protocol by including multi-phase CM-enhanced full-body scans has been introduced at our emergency radiology unit. The primary aim of this study was to investigate if the newly developed low-dose multi-phase trauma WBCT protocol on Revolution™ is superior in potentially critically injured trauma patients with respect to diagnostic accuracy, when compared to trauma patients who are examined with our standard single-phase trauma protocol on the neighbored 64-slice LightSpeed VCT. Our secondary aims were to evaluate and compare the radiation dose, image quality, and workflow between the two trauma CT protocols.

## Material and methods

This study has been approved by the regional ethics committee (#2016/1347-31/2).

We retrospectively analyzed 474 trauma patients who presented to the level 1 trauma unit at Karolinska University Hospital Solna between March and September 2015. During this period, both the LightSpeed™ VCT and the Revolution™ CT were concomitantly used in our clinical trauma management. Inclusion criteria were as follows: age >16 years, level 1 or 2 trauma activation, and a WBCT scan with the LightSpeed™ VCT single-phase protocol or a WBCT scan with the Revolution™ CT multi-phase low-dose protocol. Exclusion criteria included unstable patients with acute post-traumatic surgery (including surgical “abdominal packing”) before CT, examination with CT protocols other than the previously mentioned, and pediatric patients (age <16 years).

The initial sample size for the group of patients who were scanned at Revolution™ CT utilizing the low-dose multi-phase trauma CT protocol (group A) was 141. Of these, 32 needed to be excluded because they were, due to acute decisions by the referring trauma surgeon after clinical assessment, not examined according to correct CT protocol. The initial sample size for the group of patients who were scanned at LightSpeed™ VCT with the single-phase trauma WBCT protocol (group B) was 121. Of these, 11 patients were excluded because they also were not examined according to the correct CT protocol. Therefore, a total of 219 patients met the inclusion criteria. The novel Revolution™ CT multi-phase low-dose protocol comprised a native scan of the scull, midface, and cervical spine followed by a whole-body (vertex to symphysis) CT angiography and an abdomen scan in venous CM phase (Table 1).

**Table 1:** Imaging parameters for protocol A using Revolution™ CT

	Native skull	Native CS	WBCT angiography	Abdomen v.p.
kV mode	120 kV, manual	100 kV, manual	100 kV, manual	100 kV, manual
mA mode	100–420 mA	100–400 mA	150 mA, manual	90 mA, manual
ASiR-V level	50%	50%	50%	50%
Detector coverage	160 mm	40 mm	80 mm	80 mm
Rotation time	1.00 s	0.35 s	0.50 s	0.50 s
Coverage speed	–	112.5 mm/s	158.75 mm/s	158.75 mm/s
Pitch	–	0.984	0.992	0.992
Prep/group delay	0.0 s	0.0 s	Smart prep	45.0 s
Scan type	Axial	Helical	Helical	Helical

*kV* kilovolts, *mA* milliampere, *ASiR-V* adaptive statistical iterative reconstruction-V, *CS* cervical spine, *WBCT* whole-body computed tomography, *v.p*. venous phase

The LightSpeed™ VCT single-phase protocol comprised a native scan of the scull, midface, and cervical spine followed by a CM-enhanced scan of the thorax and abdomen during venous phase only (Table 2). The amount of intravenously given contrast (1.1 ml/kg) per patient did not differ between both protocols, and the same type of contrast agent was used throughout the whole study (Iohexol, Omnipaque™ 350 mg I/ml, GE Healthcare, Milwaukee, WI, USA).

**Table 2:** Imaging parameters for protocol B using LightSpeed™ VCT

	Native skull	Native CS	Thorax + abdomen v.p.
kV mode	120 kV	120 kV	120 kV
mA mode	330–350 mA	100–400 mA	150–550 mA
Detector coverage	20 mm	20 mm	40 mm
Rotation time	0.6 s	0.5 s	0.4 s
Coverage speed	17.7 mm/s	38.74 mm/s	137.5 mm/s
Pitch	0.531	0.969	0.984
Prep/group delay	0.0 s	0.0 s	55.0 s
Scan type	Helical	Helical	Helical

*kV* kilovolts, *mA* milliampere, *CS* cervical spine, *v.p.* venous phase

Mean Houndsfield unit (HU) values with standard deviation (SD) and signal-to-noise ratios (SNRs) of the scans during native scull, native cervical spine, and portal venous phase for each of the two CT machines were measured using circular region of interests (ROIs) placed in the brain, cervical spine, abdominal aorta, and liver, respectively. The ROI placement was standardized. The ROI for the brain was placed in the white matter of the centrum semiovale. The ROI for the cervical spine was placed intraspinally at the level of the fourth cervical bone (C4), the ROI for the abdominal aorta was below the level of the diaphragm, and the ROI for the liver was placed in segment 8. To reduce measurement bias, ROIs were kept constant for each body part and at 20 mm^2^ for the liver, 12 mm^2^ for the abdominal aorta, 10 mm^2^ for the cervical spine, and 15 mm^2^ for the brain. Artifacts, blood vessels, and calcifications were carefully excluded from the ROI measurement areas. Thus, to encompass homogeneity of measured tissues, the ROI area varied from 12 to 20 mm^2^. The SNR was calculated as the HU value of the ROI divided by one SD of the ROI.

To measure DICOM images and to determine mean CT values and SD for all pixels in each ROI, the ImageJ™ software (National Institutes of Health, USA; http://imagej.nih.gov.ij) was used.

Two radiologists with board certificate recognized in the European Union independently retrospectively analyzed 50 scans from group A and 50 scans from group B. The radiologists were blinded to CT parameters and patient clinical details. Images were semiquantitatively assessed for quality according to the European Guidelines on Quality Criteria for CT and graded on a four-point scale where score 4 was given for excellent quality, 3 for good quality, score 2 if quality was poor but sufficient for diagnostic evaluation, and 1 if image quality was insufficient for diagnostic evaluations.

Values of dose length products (DLP; mGy*cm) were provided by the CT scanners, and the effective dose (ED; mSv) for each examination was calculated and compared between the two trauma CT protocols.

The definitive radiology reports of the trauma CT examinations were retrieved and reanalyzed for this study. At our institution, all radiology examinations are assessed for a preliminary report (signature 1) by the respective radiologist on duty. Thereafter, all reports are reassessed, if necessary corrected/changed, and signed (signature 2) as the definite report by a consultant. For this study, we reassessed all emergency trauma CT examinations a third time regarding types and frequencies of trauma-related findings. In addition, we analyzed the frequency of missed injuries in the preliminary CT report when compared to the definite report and the third assessment in the course of this present study. Missed injuries were defined as trauma-related findings that were present on admission, but not described in the preliminary trauma CT report. Missed injuries were divided into perceptual or interpretational errors. Perceptual errors resulting from not recognizing findings can be influenced by image quality, technical, or methodical limitations. Interpretational errors result from not recognizing the implications or misinterpreting findings and are often due to lack of knowledge/experience. Of note, the purpose of this evaluation was not to analyze the performance of preliminary readers.

Furthermore, discrepancies between trauma-related arterial injuries in preliminary reports, definitive reading, and reassessment were classified into true positive, false positive, true negative, and false negative with consecutive calculation of the sensitivity, specificity, positive predictive value, and negative predictive value. The reference standard was defined as a combination of all available patient-journal follow-up data for each patient including clinical, laboratory, imaging, and, if applicable, surgical/interventional data. A finding was considered true positive if a radiologically diagnosed arterial injury was confirmed by follow-up data. If no trauma-related arterial injury was found and follow-up data was unremarkable, findings were considered true negative. If trauma CT indicated something potentially clinically important, which follow-up data could not prove, the finding was considered false positive. If the radiologist did not report an arterial injury that follow-up data showed, it was considered false negative. In addition, discrepancies in the incidence of trauma-related non-arterial injuries between the two groups were assessed.

Finally, we evaluated if patients from group B needed to undergo a protocol modification during trauma CT and complementary CT angiography of the cervical spine, which was not part of their standard trauma CT protocol, was requested.

The mean time from patient registration at the CT until completion of examination and from completion of examination until radiologist’s preliminary written report was recorded and compared for all patients and used for between-group comparisons.

### Statistical analysis

Statistical analysis was performed using the software IBM SPSS (version 22, Chicago, IL, USA). Formal descriptive statistical analysis was used for between-group comparisons. The unpaired *t* test was used to compare variables with normal distribution, the chi-squared test for dichotomous variables, and the Mann-Whitney *U* test for ordinal and continuous variables with non-normal distribution. Cohen’s kappa was used to assess interrater agreement, when a *κ*-value of <0 was considered no agreement, a *κ*-value <0.20 poor agreement, a *κ*-value of 0.21–0.40 fair agreement, a *κ*-value of 0.41–0.60 moderate agreement, a *κ*-value of 0.61–0.80 good agreement, and a *κ*-value of 0.81–1 very good agreement. The level of significance was *p* ≤ 0.05.

## Results

The mean (±SD) age of patients from group A (26 females/83 males) was 44 years (19) and did not statistically significantly differ from patients of group B [46 years (20); 37 females/73 males; *p* = N.S.].

**Fig. 1:**
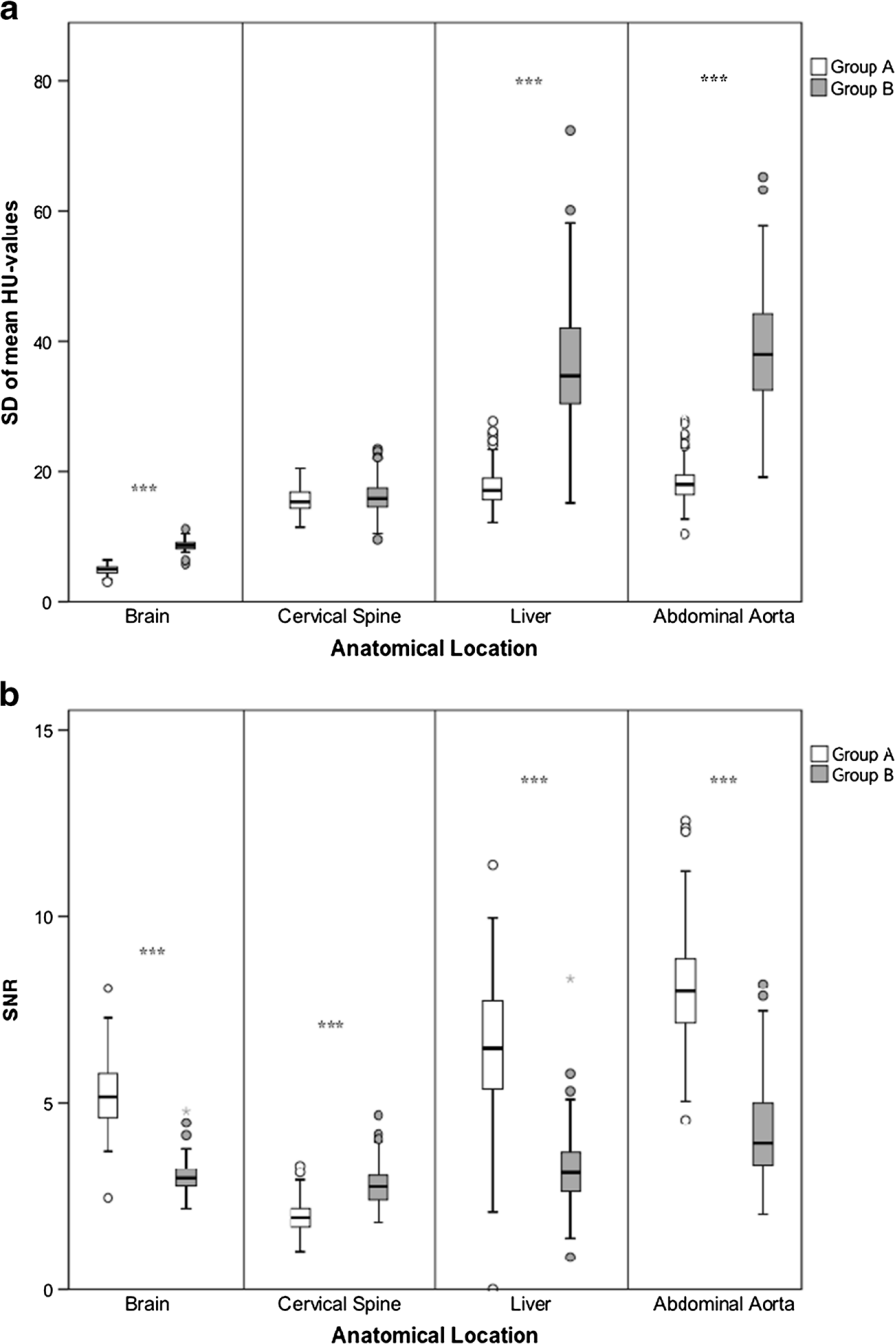
Boxplot diagram showing **a** the mean SD of the mean HU values and **b** SNR for the brain (B), cervical spine (CS), liver (L), and abdominal aorta (AA). *Boxes* represent the middle 50% of the data, and *solid lines* represent the median. The *whiskers* represent the minimum and maximum values. *Circles* represent outliers and *stars* represent extreme outliers. ***p < 0.001

The SDs of the mean HU values of the brain, liver, and abdominal aorta, but not cervical spine, were statistically significantly higher in group B when compared to group A (*p* < 0.001; Fig. 1a). Mean SNRs for the brain, liver, and abdominal aorta were statistically significantly higher in group A than in group B (*p* < 0.001; Fig. 1b). However, the mean SNR for the cervical spine was significantly higher in group B when compared to group A (*p* < 0.001; Fig. 1b).

**Fig. 2:**
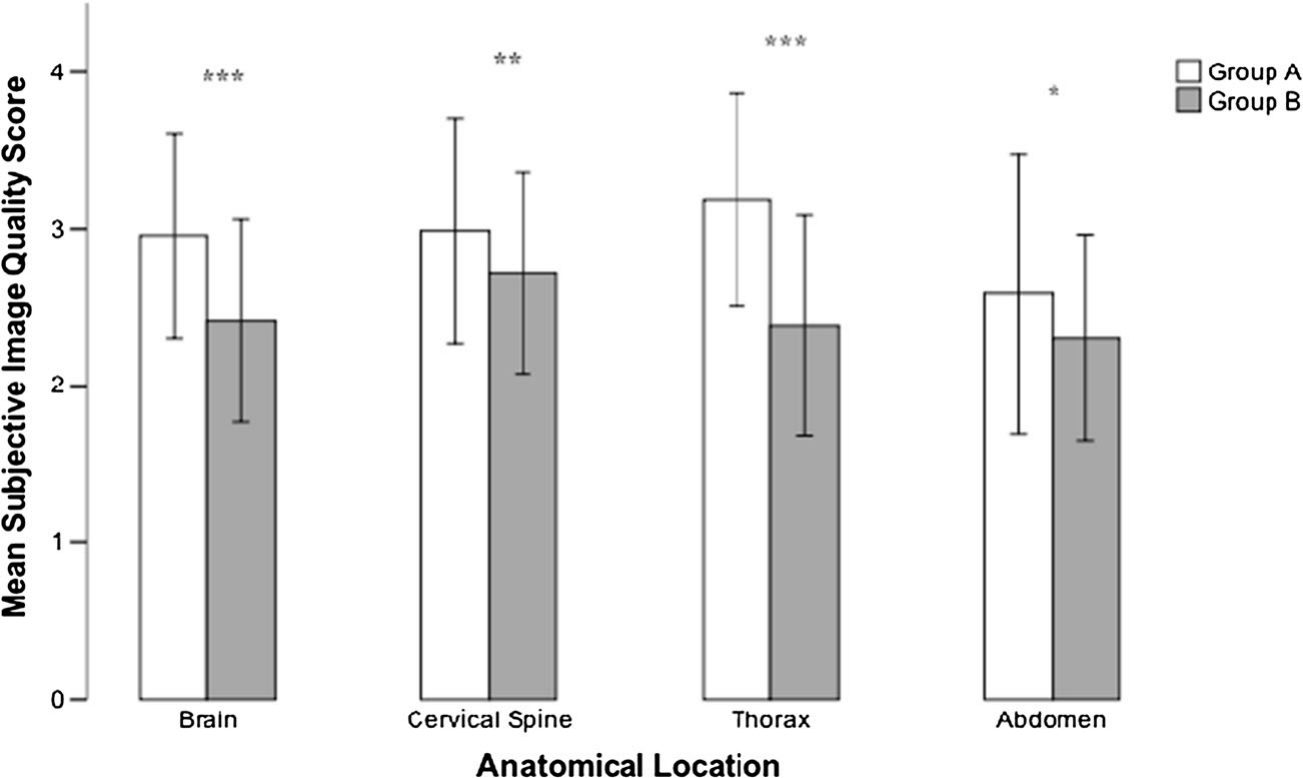
Bar graphs showing the mean subjective image quality score (±SD) for the brain, cervical spine, thorax, and abdomen. Group A had statistically significantly higher image quality scores for all analyzed body parts (*p* values varying from ***p < 0.001 to *p < 0.015)

The results of semiquantitative subjective scoring of image quality for different anatomical parts revealed that the image quality scores for all analyzed body parts were assessed with statistically significantly higher mean scores for group A (Fig. 2; *p* < 0.01, respectively). The lowest scores for both groups were found for image quality of the abdomen (2.6 ± 0.9 for group A vs. 2.3 ± 0.7 for group B). The greatest between-group difference was found for the image quality of the thorax (3.2 ± 0.7 for group A vs. 2.3 ± 0.7 for group B; *p* < 0.001). The *κ*-values for the blinded semiquantitative assessment of image quality for the two radiologists ranged from 0.60 to 0.81 (mean 0.67), which indicates high agreement.

Group A had a mean DLP of 1681 ± 183 mGy*cm, which was statistically significantly lower that the DLP of group B (1932 ± 247 mGy*cm; *p* < 0.001). In other words, protocol A gave a dose reduction of 13.0%. Analogous mean ED was 11.5 ± 1.5 mSv in group A and 11.8 ± 3.1 mSv in group B (*p* = N.S.).

A detailed summary of the most frequent types of injuries is shown in Table 3. In group A, five BCVIs were detected, whereas no BCVI was detected in group B (*p* = 0.03; Table 3). Overall, using CT protocol A, statistically significantly more arterial dissections and arterial injuries could be identified when compared to protocol B (*p* < 0.04; Table 3).

**Table 3:** Traumatic injuries identified by trauma CT protocol in group A and group B

Type of injury	Group A (*n* = 109)	Group B (*n* = 110)	*p* value
Head/neck			
I.C. hemorrhage	28 (11.8)	23 (10.5)	0.645
Midface	21 (8.9)	21 (9.5)	0.800
Skull	20 (8.4)	23 (10.5)	0.461
Abdomen			
Spleen	3 (1.3)	4 (1.8)	0.631
Liver	6 (2.5)	2 (0.9)	0.186
Kidneys	3 (1.3)	1 (0.5)	0.352
Adrenals	2 (0.8)	–	0.172
Pancreas	2 (0.8)	–	0.172
Bowel/mesentery	–	2 (0.9)	0.141
Thorax			
Ribs	23 (9.7)	28 (12.7)	0.305
Pleural space	16 (6.8)	21 (9.5)	0.274
Lungs	14 (5.9)	22 (10.0)	0.105
Scapula	4 (1.7)	4 (1.8)	0.915
Clavicles	8 (3.4)	5 (2.3)	0.479
Sternum	6 (2.5)	3 (1.4)	0.369
Mediastinum	6 (2.5)	3 (1.4)	0.369
Pelvis			
Pelvic girdle	13 (5.5)	7 (3.2)	0.229
RPH	6 (2.5)	4 (1.8)	0.602
Spine			
Lumbar	11 (4.6)	8 (3.6)	0.591
Thoracic	9 (3.8)	13 (5.9)	0.292
Cervical	6 (2.5)	11 (5.0)	0.164
Upper extremity	4 (1.7)	–	0.053
Lower extremity	5 (2.1)	6 (2.7)	0.667
Active arterial bleeding	11 (4.6)	8 (3.6)	0.591
Kidney	1 (0.4)	1 (0.5)	0.958
Spleen	1 (0.4)	–	0.335
Liver	1 (0.4)	–	0.335
Lung	4 (1.7)	–	0.053
Pelvis	3 (1.3)	1 (0.5)	0.352
Aortic branches	–	1 (0.5)	0.299
Thoracic wall	–	1 (0.5)	0.299
Mesentery	–	2 (0.9)	0.141
Muscles/s.c. tissue	1 (0.4)	2 (0.9)	0.519
BAI	3 (1.3)	1 (0.5)	0.352
Intramural hematoma	1 (0.4)	–	0.335
Intimal tear	1 (0.4)	–	0.335
Pseudoaneurysm	1 (0.4)	–	0.335
Rupture	–	1 (0.5)	0.299
BCVI	5 (2.1)	0	0.030
Carotid	4 (1.7)	0	0.053
Vertebral artery	1 (0.4)	0	0.335
Other	2 (0.8)	0	0.172
Subclavian artery	2 (0.8)	0	0.172
Arterial dissections, total	10 (4.2)	0	0.002
Arterial injuries, total	21 (8.9)	9 (4.1)	0.040

Values are given as total numbers of injuries (percentage of the total number of all injuries per group)

*I.C.* intracranial, *RPH* retroperitoneal hemorrhage, *s.c.* subcutaneous, *BAI* blunt aortic injury, *BCVI* blunt cervical vascular injury

Analysis of discrepancies between trauma-related arterial injury findings from the preliminary report and the definitive reading showed that 7 out of 21 arterial injuries were primarily missed with protocol A, corresponding to a sensitivity of 67%. All of the missed arterial injuries were, however, picked up on the definitive reading, corresponding to a total sensitivity of 100%. There was one false-positive finding after definitive reading, where an additional DT angiography confirmed a pulsation artifact and not an arterial dissection. In total, this corresponds to a specificity of 99%, a positive predictive value of 95%, and a negative predictive value of 100% with protocol A, after definitive reading. Four of 12 arterial injuries were missed with protocol B with no differences between preliminary and definitive readings, corresponding to a sensitivity of 67%, a specificity of 100%, a positive predictive value of 100%, and a negative predictive value of 96% after definitive reading.

There was no statistically significant difference between the incidences of missed non-arterial injuries in the preliminary report between the two protocols, with respect to non-arterial diagnoses. Reevaluation of the missed injuries showed a total number of four perceptual errors with protocol A: one intraventricular bleeding in the dorsal horn of the lateral ventricle that was hard to detect due to streak artifacts, one undisplaced rib fracture, one fracture of the transverse process of a lumbar vertebra, and one pancreatic tail fracture. There were two perceptual errors with protocol B: 1 rib fracture and 1 compression fracture of a thoracic vertebra. All other initially missed injuries were considered to be due to interpretational errors only.

In group B, nine patients (8%) had to undergo a complementary CT angiography of the cervical vessels, and in four of these patients, an arterial injury was detected. The findings during CT according to protocol B that lead to the decision for a complementary CT-angiography were fractures (*n* = 7) and extensive intracranial hemorrhages (*n* = 2). Of note, of five BCVIs that were detected with trauma CT protocol A, only two showed concomitant fractures (in both cases, a fracture involving the carotid canal with associated ICA dissection). The three remaining BCVI (a left ICA dissection, a left CCA dissection, and a left VA dissection) did not have any associated fracture.

The mean (±SD) time from patient registration until completion of examination was statistically significantly longer for group A compared to group B (27.8 ± 16 vs. 20.8 ± 15 min; *p* < 0.001). The mean (±SD) time from completion of examination until the preliminary written radiology report was 46.6 ± 27 min for group A and 48.3 ± 38 min for group B (*p* = N.S.). Mean (±SD) total time from patient registration until preliminary written radiology report was 75.1 ± 34 min for group A and 69.4 ± 41 min for group B (*p* = 0.03).

## Discussion

We systematically analyzed a trauma CT protocol that combines multiple CM phases together with the latest dose reduction techniques. Thus, CT protocol A is now the routinely used trauma CT protocol at the Emergency Radiology Unit radiology, Karolinska University Hospital (blinded), and this project has therefore been part of a necessary quality control study. The purpose of the new trauma CT protocol is to increase the diagnostic accuracy for trauma-related arterial injuries because of their high rate of mortality and the need for a prompt and accurate diagnosis. Concomitantly, we wish to increase the additional diagnostic benefits of a multi-phase scan without the need of a higher total radiation dose or delays in workflow. Finally, we need to ensure that the latest dose reduction techniques, together with the, at present, most advanced CT hardware, provide the most satisfying image quality for our critically injured patients.

This study, together with several others [18–20], showed that the addition of an arterial phase in the trauma CT protocol improves the diagnostic accuracy for arterial injuries. For definitive reports, sensitivity, positive, and negative predictive values were almost at 100%. Preliminary reporting yielded a lower sensitivity of 67%, which mainly might be reasoned by an initial unfamiliarity of our on-site radiologists to a trauma CT protocol that includes a WBCT angiography. This theory is further supported by the observation that all of initially overlooked arterial injuries have been detected during secondary definitive reading by our most experienced trauma radiologists. This is also in line with other studies that reported a stronger sensitivity with new CT protocols after definitive readings [21]. We believe that with increasing time and routine, all our radiologists will develop further experience in reading trauma CT examinations according to the low-dose multi-phase protocol, and thus, the diagnostic sensitivity will increase. Previously, a complementary CT angiography has only been performed when the suspicion of arterial injuries was very high, based on the clinical presentation or visible injuries suspected for active bleedings. Further evaluation of this routine showed that the decision for a complementary CT angiography was exclusively based on the detection of fractures (7 of 9) or the detection of extensive intracranial hemorrhages. In this context, it needs mentioning that only 2 of the 5 BCVIs detected with trauma CT protocol A showed an associated fracture. This is in concordance with previous studies that demonstrated that in a significant number of cases, BCVI can be found without associated fractures [15, 22].

Our low-dose multi-phase trauma CT protocol uses a single contrast injection for WBCT angiography followed by an abdominal scan in venous phase. Compared to a requested complementary CT angiography, this method saves time and does not increase the intravenous contrast load. It has been shown that the request for a complementary CT angiography leads to a risk of delayed diagnoses in the emergent situation [20].

We also assessed the incidence of missed non-arterial injuries in both groups and did not find a statistically significant difference. Therefore, the diagnostic accuracy of trauma-related non-arterial injuries is not affected by the low-dose multi-phase trauma CT protocol. Among the most frequently missed injuries in both groups were fractures of the ribcage, the midface, and the transverse vertebral processes. Similarly, several others have shown that the most frequently missed diagnoses during a trauma CT are skeletal injuries [21, 23]. This has been reasoned with the explanation that, e.g., broken nasal bones and fractured ribs do not affect the emergency handling of a potentially critically injured patient and that the radiologists purposely direct their main attention to obvious and vitally important injuries, such as arterial or organ injuries, mostly only found during the arterial phase of a CT scan [21, 23].

Radiologists are responsible for keeping the radiation doses of their patients as low as possible, especially with the increasing demand for CT examinations, even in emergency situations. It has been reported that the latest iterative reconstruction technique ASIR-V can lower the radiation dose up to 82%. Therefore, we initially hypothesized that the addition of a WBCT angiography to the routinely used trauma CT protocol will, compared to our old CT protocol, result in at least equal or most likely even lower total radiation dose. The results of this present study showed that the DLP was significantly lower with the new trauma CT protocol and corresponding ED values did not statistically significantly differ between the two groups. We also noticed that the WBCT angiography was responsible for at least half of the total radiation dose in CT protocol A. Both protocols presented DLP and ED values that were within the normal range for a trauma CT. Therefore, the inclusion of a WBCT angiography in the low-dose multi-phase trauma CT protocol is ethically fair with respect to radiation levels. Excluding the WBCT angiography would most likely lower at least half of the total radiation dose but simultaneously increase the risk of overseeing life-threatening trauma-related arterial injuries. Hence, we believe that the low-dose multi-phase trauma CT protocol represents a satisfactory balance between additional diagnostic value for trauma-related injuries and total radiation dose.

For trauma protocol A, both the time from patient registration until completion of examination and the time from patient registration until radiologist’s preliminary written report took significantly longer. This was expected due to adding the CT angiography, and this result was in line with other studies [24, 25]. For acute emergency situations, when time is one of the most relevant factors, additional technology can sometimes, like in our case, reduce the workflow due to longer post-processing and image transfer times. Thus, due to technical complexities and surplus electronic data provided by the newer low-dose technique, examination time increases. As mentioned, this study retrospectively investigated all examinations performed between March and September 2015. After the study period, a software update package has been installed that improved data acquisition and image transfer times and this was noticed in the clinical work; further studies are, however, needed to verify this.

There was no statistically significant difference between the two groups for time from completion of examination until radiologist’s preliminary report. This finding is interesting since we expected to find a significantly longer interpretation time for the low-dose multi-phase trauma CT protocol A because of almost twice as much diagnostic imaging load and on-site colleagues that are not too familiar with reading WBCT angiographies. We are satisfied to know that the increased total time for group A is mainly due to extra post-processing and image transfer time and not due to prolonged interpretation time. We also expect the total time to shorten due to the fact that the radiologists and technicians will get accustomed to the new CT protocol. A pilot study, which was conducted before this present study, confirmed this hypothesis, and we saw a gradual decrease in total examination and interpretational time for consecutive patients (unpublished data).

With the low-dose multi-phase trauma CT protocol, we found significantly lower SD from mean HU values and a significantly higher SNR for the brain, liver, and aorta. However, there was no statistically significant difference in SD from mean HU values for the cervical spine and there was even a statistically significant higher SNR with trauma CT protocol B. This finding was not expected and could be due to misplacement of ROI circles. ROIs were, as mentioned, placed manually in respective anatomical locations. The cervical spinal canal is a very confined area surrounded by dense bone structures. If the ROI circle only included a few pixels of surrounding bone, a deterioration of mean HU values and registration of extreme values could occur. Some of the extreme values were probably due to this phenomenon, especially in patients with anatomically narrow spinal canal. We could have analyzed the cervical spine with smaller ROIs, but this also comes with the cost of less reliable mean HU values.

Another aspect that could explain the unexpected cervical spine quality statistics is the fact that potentially critically injured trauma patients often arrive with a cervical collar and are often intubated. These objects can cause artifacts and alteration of the image quality.

Semiquantitative image quality assessment showed significantly higher image quality scores for all anatomical locations with trauma protocol A. The difference in scores was the least for the cervical spine, supporting the quantitative image quality finding that there was no significant difference in SD from mean HU values.

There are several strengths with our study. First, it includes relatively large sample sizes. Second, the two different CT machines with their respective trauma CT protocols were simultaneously used at our Radiology department and the patients were examined on either the Revolution™ CT or the LightSpeed™ VCT. The decision to which machine the trauma patients were transferred was solely based on their availability. This, partially, makes this study randomized and allows for reliable between-group comparisons. No other factors, such as, e.g., level of trauma or experience of personal, influenced the choice of CT machine for trauma patients.

It has to be mentioned, however, that our study also had a number of limitations. The two different trauma CT protocols were not compared in the same patients since the study was performed retrospectively in a real-life clinical setting. Moreover, a comparison between the diagnostic performances is hard when the extent and location of trauma-related injuries naturally differ from one patient to another. Another limitation of this retrospective study is that the trauma CT scans were not obtained with the same machine. Thus, we are lacking an ideal control group since differences in scanner attributes could have affected the results. The goal of our study was, however, not to perform an isolated evaluation of a reconstruction algorithm from one manufacturer. We wished to evaluate our standard single-phase trauma CT protocol against a new low-dose multi-phase trauma CT protocol and to determine if it is clinically feasible, delivers accurate diagnostic imaging, and reduces the RD. While a comparison made with the same machine would also have provided important insights, this study reflects the real-life situation of an advanced level 1 trauma center where, like in many other trauma centers worldwide, acute trauma CT scans are obtained at one site on different scanners from different manufacturers. Even before this study, our department’s expectations were high on the performance of latest generation of this 16-cm detector Revolution™ MDCT with the latest iterative reconstruction techniques. This quality reassurance study in large parts met those high expectations. We managed to show that our low-dose multi-phase trauma CT protocol reduced radiation doses despite the inclusion of additional CM-enhanced series. The protocol proved to have markedly improved the diagnostic accuracy for trauma-related arterial injuries and also image quality. Therefore, the new trauma CT protocol is now used as the standard protocol in all trauma level 1 and 2 patients and fully integrated in our early management of potentially critically injured trauma patients. Moreover, from personal communications, we know that this protocol is already now, due to its proven higher diagnostic value and benefit for the patients, widely accepted among both doctors and radiology technologists.

Our study has provided important information that could be further investigated in future studies. As on-site radiologists and radiology technicians get more accustomed to the new trauma CT protocol, we expect an even better performance in preliminary report diagnostic accuracy and examination time, respectively. This needs to be evaluated and confirmed with future studies. Further optimization of imaging parameters and development imaging reconstruction techniques is a constantly continuous process and interesting substance for future studies.

Despite the inclusion of additional CM-enhanced series in the CT protocol, we have managed to keep radiation doses below the normal range of a trauma CT. However, the vast amount of electronic information has led to a slight but significant increase in examination time, which could lead to a reduction in workflow for acute emergency situations when time is a most relevant factor.

## Conclusion

To our knowledge, this is the first trauma CT protocol that combines multiple CM phases together with the latest dose reduction techniques, such as ASIR-V, in a clinical trauma setting. Despite the inclusion of additional CM-enhanced series in the CT protocol, we have managed to keep radiation doses below the normal range of a trauma CT. The protocol has markedly improved the diagnostic accuracy for trauma-related arterial injuries that otherwise could have been missed and also improved image quality. However, it needs to be mentioned that, due to technical complexities and surplus electronic data provided by the newer low-dose technique, examination time increases.
